# Supplementary material for: Coenzyme A is a redox sensing cofactor for malic enzyme 2 regulating oxidative stress and mitochondrial metabolism
Source: bioRxiv. 2026 Apr 28:2026.04.27.721221. Preprint. [Version 1] doi: 10.64898/2026.04.27.721221 (PMC13142473; doi:10.64898/2026.04.27.721221)
Supplement: Supplement 1 [file NIHPP2026.04.27.721221v1-supplement-1.pdf]

bioRxiv preprint doi: https://doi.org/10.64898/2026.04.27.721221; this version posted April 28, 2026. The copyright holder for this preprint (which was not certified by peer review) is the author/funder, who has granted bioRxiv a license to display the preprint in perpetuity. It is made available under aCC-BY 4.0 International license.

Supplementary Figures

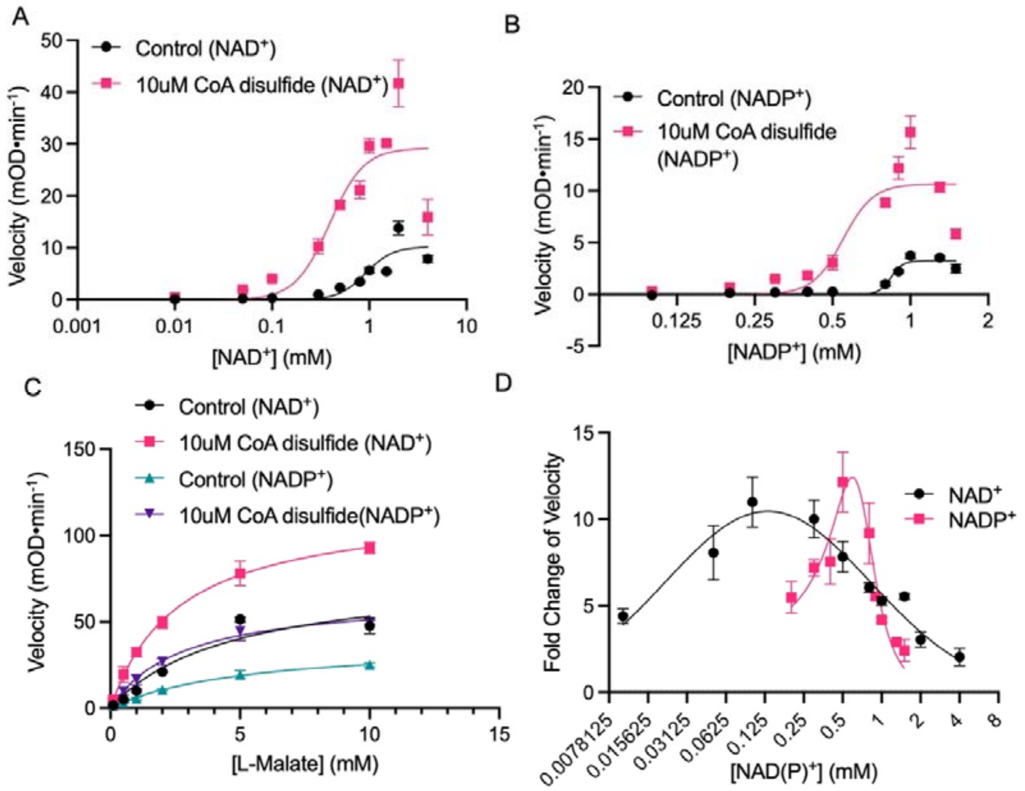

Figure S1. ME2 enzyme activity under various substrate concentrations. ME2 enzymatic activity with and without CoA disulfide was measured at various (A) NAD^+, (B) $NADP^+$, (C) L-malate concentrations while keeping other substrate concentration constant. (D) CoA disulfide promotes both NAD^+ and $NADP^+$-dependent ME2 enzyme activity, but the degree of activation varies at different nucleotide concentrations.

bioRxiv preprint doi: https://doi.org/10.64898/2026.04.27.721221; this version posted April 28, 2026. The copyright holder for this preprint (which was not certified by peer review) is the author/funder, who has granted bioRxiv a license to display the preprint in perpetuity. It is made available under aCC-BY 4.0 International license.

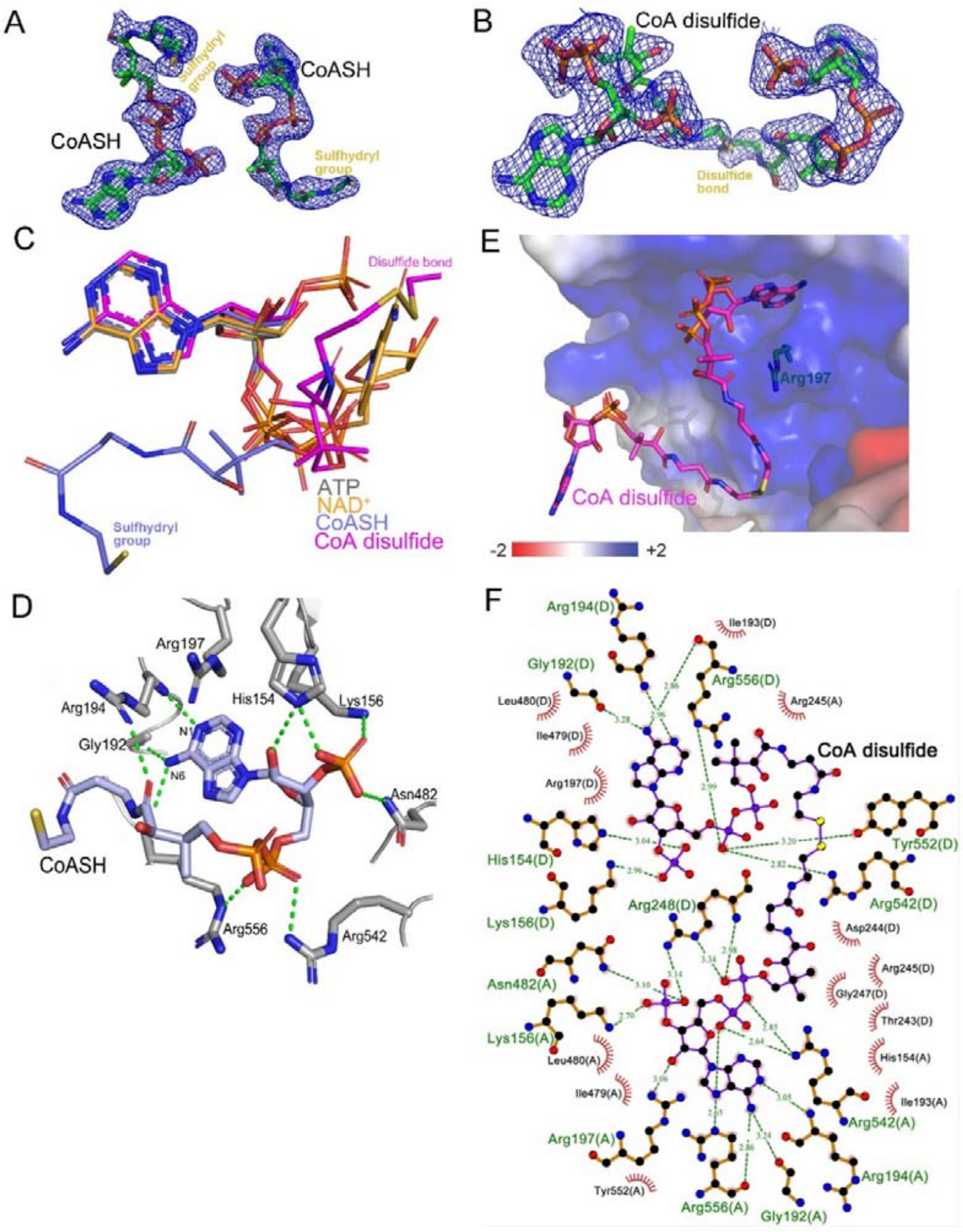

Figure S2. **Structural analysis of CoASH and CoA disulfide binding to ME2 and ligand comparison at the exo site.** *2Fo-Fc* electron density maps for the two CoASH molecules of ME2-CoASH complex structure (A) and a CoA disulfide molecule of ME2-CoA disulfide complex structure (B). The electron density is displayed as a blue mesh (contoured at 1.0 σ). (C) Schematic drawing showing the overlay of the ATP (gray), NAD^+ (orange), CoASH (slate) and CoA disulfide (magenta) bound at the exo site of ME2. (D) The binding site of CoASH in the ME2-CoASH complex structure. CoASH (slate) and interacting protein residues (gray) are shown as sticks. Hydrogen bonds between the protein and the cofactor are indicated as green dotted lines. (E) The surface potential of the ME2-CoA disulfide complex generated by the APBS tool of

bioRxiv preprint doi: https://doi.org/10.64898/2026.04.27.721221; this version posted April 28, 2026. The copyright holder for this preprint (which was not certified by peer review) is the author/funder, who has granted bioRxiv a license to display the preprint in perpetuity. It is made available under aCC-BY 4.0 International license.

PyMOL (unit: kT/e) was displayed as a color gradient ranging from red (negative) to blue (positive). Detailed interactions and hydrogen bonds between ME2 and CoA disulfide in the ME2-CoA disulfide binary complex structure (F) are shown as the LigPlot diagram, note that different chains were labelled in the parentheses. The green dashed lines correspond to the hydrogen bonds. Spoked arcs represent hydrophobic contacts.

bioRxiv preprint doi: https://doi.org/10.64898/2026.04.27.721221; this version posted April 28, 2026. The copyright holder for this preprint (which was not certified by peer review) is the author/funder, who has granted bioRxiv a license to display the preprint in perpetuity. It is made available under aCC-BY 4.0 International license.

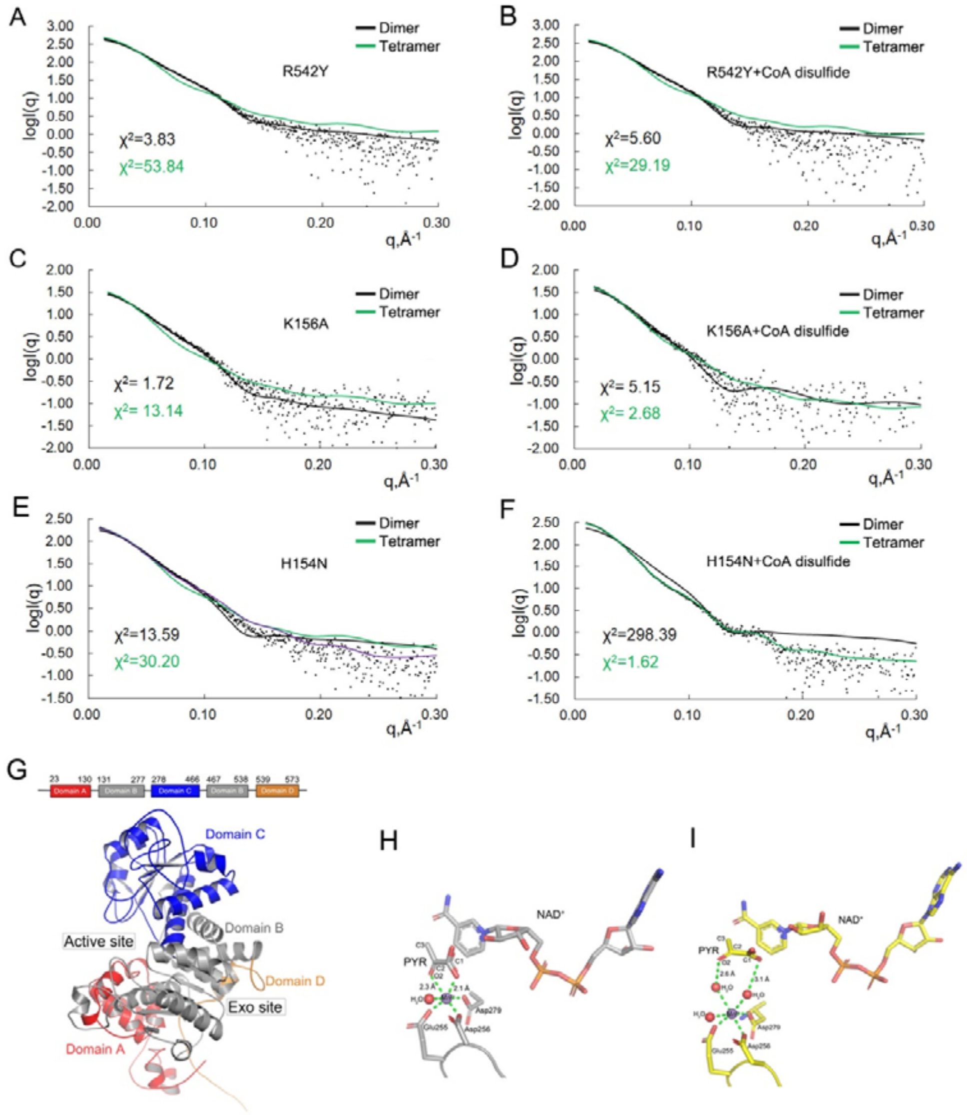

Figure S3. SAXS analysis of ME2 mutants reveals oligomeric state in the presence of CoA disulfide and structural comparison with ME2-CoASH complexes. Overlay of experimental scattering profiles with calculated scattering profiles for R542Y (A, B), K156A (C, D) and H154N (E, F) mutants, alone and in the presence of CoA disulfide. Experimental data are represented in black dots. The theoretical scattering curves of dimers (black lines) and tetramers (green lines) are shown. (G) Schematic domain and a cartoon representation of the apo-ME2 protomer. The domains A, B, C and D of the structure are shown in red, gray, blue and brown, respectively. The active site and exo site of the protomer are also indicated. Schematic drawing of the active site in the crystal structure of ME2 in complex with CoA disulfide/NAD^+/PYR/Mn^{2+} (H) and CoASH/NAD^+/PYR/Mn^{2+} (I). The manganese ion and the ligated water are shown as spheres.

bioRxiv preprint doi: https://doi.org/10.64898/2026.04.27.721221; this version posted April 28, 2026. The copyright holder for this preprint (which was not certified by peer review) is the author/funder, who has granted bioRxiv a license to display the preprint in perpetuity. It is made available under aCC-BY 4.0 International license.

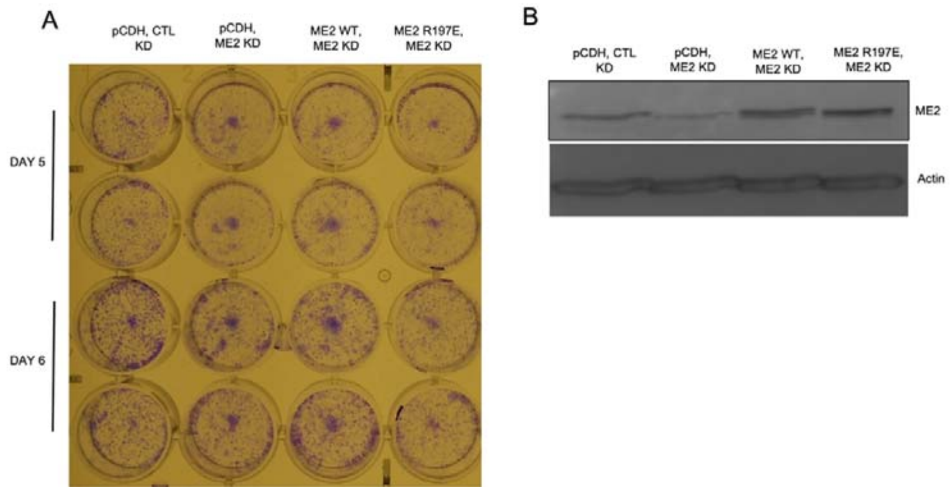

Figure S4. The effect of ME2 R197E on A549 cells colony formation ability. A549 control knockdown and ME2 knockdown cells were stably transfected with pCDH, ME2 WT, or ME2 R197E as indicated. Colony formation assay was performed to measure the tumorigenesis effect on ME2 knockdown and ME2 WT or R197E re-expression. (A) Representative images of colony formation assay at day 5 and 6; (B) Western blot for the validation of ME2 knockdown and ME2 WT or R197E re-expression.

bioRxiv preprint doi: https://doi.org/10.64898/2026.04.27.721221; this version posted April 28, 2026. The copyright holder for this preprint (which was not certified by peer review) is the author/funder, who has granted bioRxiv a license to display the preprint in perpetuity. It is made available under aCC-BY 4.0 International license.

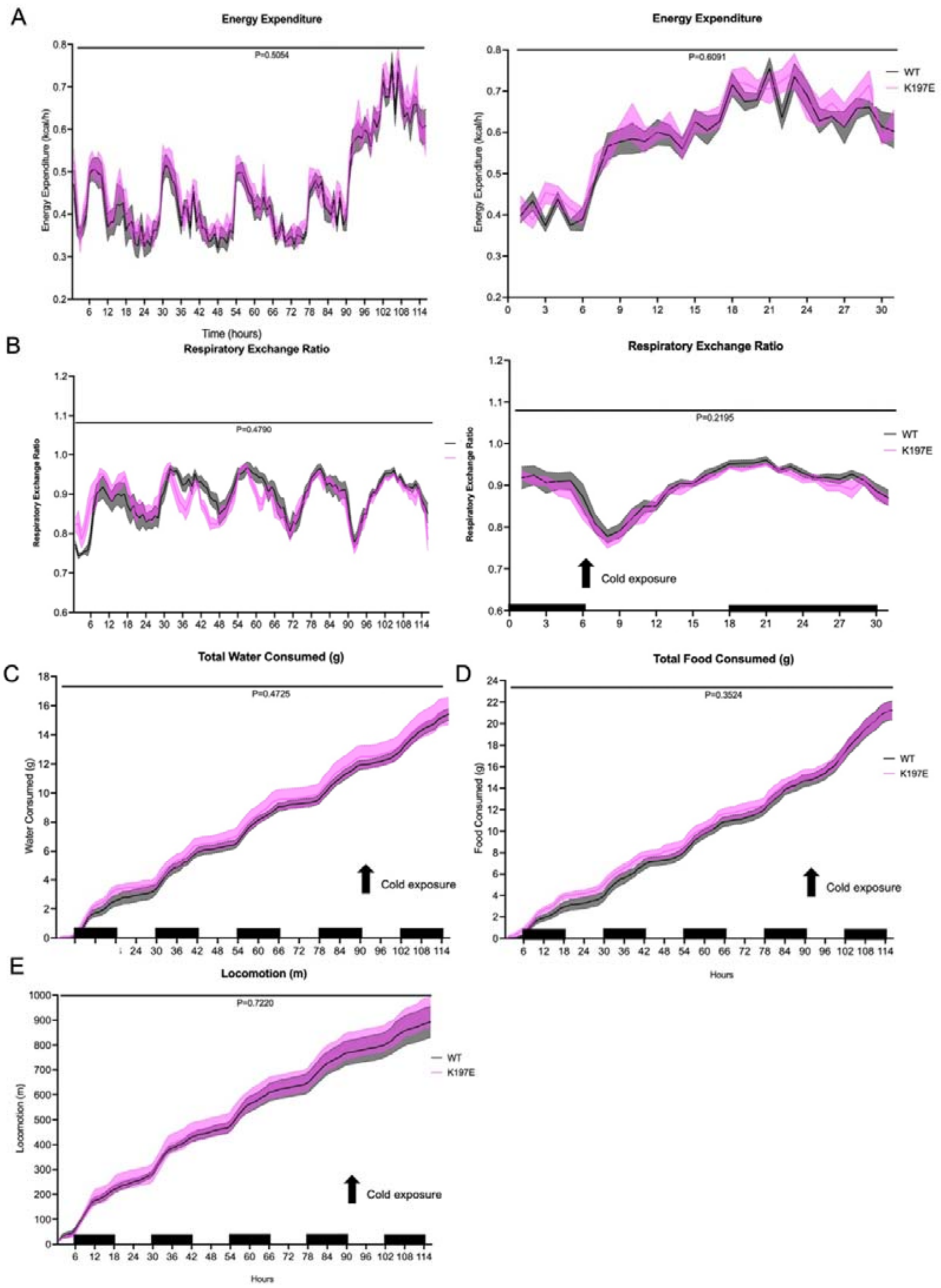

Figure S5. Metabolic cage result for *Me2 WT* (gray) and *K197E* mice (purple). The *Me2 K197E* CRISPR knock-in mice were generated on a C57BL/6J background and subject to a metabolic cage experiment. (A) Energy expenditure, (B) Respiratory exchange ratio, (C) Total water consumption, (D) Total food consumption and (E) Locomotion were monitored for 114 hours.

bioRxiv preprint doi: https://doi.org/10.64898/2026.04.27.721221; this version posted April 28, 2026. The copyright holder for this preprint (which was not certified by peer review) is the author/funder, who has granted bioRxiv a license to display the preprint in perpetuity. It is made available under aCC-BY 4.0 International license.

Supplementary Table 1. Statistics of SAXS analysis

(*a*) Sample details.

Protein	ME2
Organism	*Homo sapiens (Human)*
Source	*E. coli* expressed
UniProt sequence ID<br>(residues in construct)	P23368<br>(18-584)
Extinction coefficient [A_{280}, 0.1%(*w*/*v*)]	0.883
Molecular mass *M* from chemical composition (kDa)	63.6
concentration (mg ml^{-1})	1.0
$\bar{\upsilon}$ from chemical composition (cm^3 g^{-1})	0.742
Particle contrast from sequence and solvent constituents, $\Delta\bar{\rho}$ ($\rho_{protein}$ − $\rho_{solvent}$; 10^{10} cm^{-2})	2.76 (12.23-9.47)
Solvent	20 mM Tris-HCl pH 7.4, 150 mM KCl, 1 mM DTT

(*b*) SAXS data-collection parameters

Instrument/data processing	BL19U2 beamline at the Shanghai Synchrotron Radiation Facility (SSRF) with Dectris PILATUS 1M detector
Wavelength (Å)	1.03
Beam size (μm)	380 (H) ×25 (V)
Camera length (m)	2.683
q-measurement range (Å^{-1})	0.008~0.45
Absolute scaling method	Comparison with scattering from 1 mm pure H_2O
Normalization	To transmitted intensity by beam-stop counter
Monitoring for radiation damage	X-ray dose maintained below 210 Gy, data frame-by-frame comparison
Exposure time	Continuous 1 s data-frame measurements
Sample temperature (°C)	10.0

bioRxiv preprint doi: https://doi.org/10.64898/2026.04.27.721221; this version posted April 28, 2026. The copyright holder for this preprint (which was not certified by peer review) is the author/funder, who has granted bioRxiv a license to display the preprint in perpetuity. It is made available under aCC-BY 4.0 International license.

(*c*) Software employed for SAXS data reduction, analysis, and interpretation.

SAXS data reduction	*RAW (Hopkins et al., 2017)*
Extinction coefficient estimate	*ProtParam (Gasteiger et al., 2005)*
Calculation of $\Delta\bar{\rho}$ and $\bar{\upsilon}$ values	*MULCh* 1.1 *(Whitten et al., 2008)*
Basic analyses: Guinier, *P*(*r*), V_P	RAW (Hopkins et al., 2017)
Atomic structure modelling	*FoXS (Schneidman-Duhovny et al., 2013)*

(d) Structural parameters

Protein	WT	WT+CoASH	WT+CoASSCoA	K156A	K156A+CoASSCoA	H154N	H154N+CoASSCoA	R197E	R197E+CoASSCoA	R542Y	R542Y+CoASSCoA	F541CY543C	F541CY543C+CoASSCoA
**Guinier analysis**													
Rg (Å)	41.52±0.36	46.29±0.18	45.24±0.14	39.92±0.36	43.06±0.57	43.77±0.15	46.04±0.12	39.54±0.20	38.74±0.14	40.08±0.19	40.55±0.23	47.08±0.11	46.26±0.08
I(0)	109.60±0.66	193.72±0.53	289.27±0.62	33.82±0.23	49.11±0.53	208.99±0.52	327.51±0.62	239.07±0.7	168.03±0.41	488.91±1.63	398.01±1.50	106.45±0.20	129.934±0.19
q_{min} (Å^{-1})	0.009	0.009	0.009	0.016	0.017	0.011	0.009	0.011	0.009	0.012	0.011	0.013	0.011
qR_g max	1.297	1.278	1.296	1.286	1.301	1.299	1.294	1.297	1.291	1.294	1.288	1.292	1.288
Coefficient of correlation, R^2	0.990	0.998	0.999	0.980	0.991	0.997	0.999	0.996	0.997	0.996	0.996	0.999	0.999
***P(r)* analysis**													
Rg (Å)	41.57±0.13	46.04±0.11	45.40±0.12	40.73±0.12	44.45±0.33	43.71±0.09	45.88±0.11	39.99±0.08	39.40±0.09	40.22±0.10	40.87±0.10	47.36±0.07	46.70±0.07
I(0)	108.70±0.43	192.70±0.40	289.40±0.56	33.93±0.12	49.91±0.35	207.30±0.38	325.30±0.54	239.00±0.56	169.00±0.34	487.50±1.17	397.90±1.10	106.10±0.12	130.10±0.15
q-range (Å-1)	0.009-0.30	0.009-0.3	0.009-0.3	0.016-0.3	0.017-0.3	0.011-0.3	0.009-0.3	0.011-0.3	0.009-0.3	0.012-0.3	0.011-0.3	0.013-0.3	0.011-0.3
Dmax	125	154	154	122	146	135	153	121	127	124	123	157	158
χ2 (total estimate from GNOM)	0.944 (0.818)	1.133 (0.930)	1.028 (0.841)	1.043 (0.880)	0.9742 (0.909)	1.191 (0.818)	1.079 (0.866)	1.124 (0.874)	0.904 (0.891)	1.139 (0.858)	1.098 (0.856)	1.153 (0.776)	1.106 (0.867)
Porod volume Vp (Å^{-3})	324000	516000	506000	310000	419000	365000	533000	304000	288000	315000	338000	502000	504000

bioRxiv preprint doi: https://doi.org/10.64898/2026.04.27.721221; this version posted April 28, 2026. The copyright holder for this preprint (which was not certified by peer review) is the author/funder, who has granted bioRxiv a license to display the preprint in perpetuity. It is made available under aCC-BY 4.0 International license.

Correlation volume Vc ($Å^{-2}$)	857.47	1178.46	1179.51	892.47	999.3	895.30	1213.19	815.60	783.67	860.67	874.8	1217.60	1179.98
**Atomistic modelling**													
Crystal structure	PDB entry dimer	PDB entry tetramer	PDB entry tetramer	PDB entry dimer	PDB entry tetramer	PDB entry trimer	PDB entry tetramer	PDB entry dimer	PDB entry tetramer	PDB entry	PDB entry	PDB entry dimer	PDB entry tetramer
q-range (Å-1)	0.009-0.3	0.009-0.3	0.009-0.3	0.016-0.3	0.017-0.3	0.011-0.3	0.009-0.3	0.011-0.3	0.009-0.3	0.013-0.3	0.011-0.3	0.013-0.3	0.011-0.3
FoXS													
χ^2	1.92	1.59	2.47	1.72	2.68	5.99	1.62	2.26	2.11	3.57	5.60	7.70	6.61
Predicted R_g (Å)	36.63	44.77	45.12	36.63	45.12	43.13	45.12	36.63	36.63	36.63	36.63	45.12	45.12
c_1, c_2	1.03, 4.00	1.00, 0.85	1.02, 0.31	1.02, 4.00	1.04, -2.00	1.03, -1.63	1.02, 1.91	1.01, 4.00	1.01, 4.00	1.03, 4.00	1.04, 4.00	0.99, -0.53	1.02, -0.09

In *FoXS*, the adjustable parameters c_1 and c_2 are adjustments for excluded volume and hydration density. c_1 can vary by 5% (0.95–1.05). c_2 is allowed to be slightly negative ($-2 \leq c_2 \leq 4$). The maximum hydration adjustment c_2 of 4.0 corresponds to ~0.388 e $Å^{-3}$ (compared with bulk solvent density ρ = 0.334 e $Å^{-3}$) and the minimum hydration adjustment c_2 of -2.0 corresponds to ~0.307 e $Å^{-3}$.

bioRxiv preprint doi: https://doi.org/10.64898/2026.04.27.721221; this version posted April 28, 2026. The copyright holder for this preprint (which was not certified by peer review) is the author/funder, who has granted bioRxiv a license to display the preprint in perpetuity. It is made available under aCC-BY 4.0 International license.

bioRxiv preprint doi: https://doi.org/10.64898/2026.04.27.721221; this version posted April 28, 2026. The copyright holder for this preprint (which was not certified by peer review) is the author/funder, who has granted bioRxiv a license to display the preprint in perpetuity. It is made available under aCC-BY 4.0 International license.

Supplementary Table 2. Data collection and refinement statistics of ME2.

	Apo (PDB 25EH)	CoASH (PDB 25EI)	CoA disulfide (PDB 25EJ)	CoASH/NAD$^+$/PYR/Mn^{2+} (PDB 25EK)	CoA disulfide/NAD$^+$/PYR/Mn^{2+} (PDB 25EL)
**Data collection**					
Wavelength	0.9792	0.9778	0.9792	0.9792	0.9792
Space group	$P2_1$	$P2_1$	$C2$	$C2$	$C2$
Cell dimensions					
a,b,c (Å)	107.94, 113.29, 109.43	106.77, 116.89, 106.94	197.26, 79.10, 169.029	204.44, 59.13, 106.91	203.88, 57.81, 144.49
α,β,γ (°)	90.00, 103.31, 90.00	90.00, 98.30, 90.00	90.00, 117.59, 90.00	90.00, 101.83, 90.00	90.00, 134.29, 90.00
Resolution (Å)†	50.00-2.50 (2.54-2.50)	50.00-2.50 (2.54-2.50)	50.00-2.90 (2.95-2.90)	52.32-2.13 (2.25-2.13)	103.43-2.32 (2.38-2.32)
Rsym (%)	11.5	9.8	5.4	7.7	6.8
I/σ	15.20 (3.30)	11.58 (2.45)	20.47 (2.08)	13.6 (2.5)	11.2 (2.1)
Completeness (%)	90.3 (57.5)	92.0 (79.6)	98.7 (99.0)	95.4 (87.1)	100.0 (99.9)
Total No. of reflections	3871391	3208240	721285	451795	356132
Unique reflections	81228	81419	50544	66939	52652
Redundancy	5.3 (2.9)	5.6 (4.5)	3.3 (3.3)	6.7 (6.7)	6.8 (6.3)
**Refinement**					
Resolution (Å)	48.24-2.48 (2.54-2.48)	48.19-2.51 (2.57-2.51)	42.26-2.90 (2.98-2.90)	50.01-2.13 (2.19-2.13)	50.01-2.32 (2.38-2.32)
No. of reflections	75409 (3929)	72351 (3981)	44814 (2311)	63571 (3364)	50232 (2393)
R_{work}/R_{free}(%)	22.13/24.19	21.57/23.44	19.08/22.72	21.00/23.61	21.64/24.50
No. Of atoms					
Protein	17070	17418	16932	8576	8657
Ligand	5	232	192	199	220
Water	435	257	35	69	112

bioRxiv preprint doi: https://doi.org/10.64898/2026.04.27.721221; this version posted April 28, 2026. The copyright holder for this preprint (which was not certified by peer review) is the author/funder, who has granted bioRxiv a license to display the preprint in perpetuity. It is made available under aCC-BY 4.0 International license.

B-factors(Å^2)					
Protein	35.84	41.58	56.56	54.45	66.70
Ligand	31.31	36.76	50.95	55.73	67.59
Water	30.67	30.29	38.48	46.25	60.79
rms deviations					
Bond lengths (Å)	0.003	0.003	0.004	0.004	0.004
Bond angles (º)	1.262	1.272	1.362	1.309	1.300
Ramachandran Plot (%)[1]	97.1/2.8/0.1	96.5/3.4/0	95.6/4.3/0.1	97.1/2.9/0	96.8/3.1/0.1

[*]Three crystal experiments for each structure.

$R_{sym}=\Sigma_h\Sigma_i|I_{h,i}-I_h|/\Sigma_h\Sigma_i I_{h,i}$, where I_h is the mean intensity of the *i* observations of symmetry related reflections of *h*.

[1]Residues in favored, allowed, and outlier regions of the Ramachandran plot.
